# The Utility of Double Opposing Transposition Flaps in the Management of Inguinal Ectopic Scrotum: Surgical Experience and Literature Review

**DOI:** 10.7759/cureus.13992

**Published:** 2021-03-19

**Authors:** Anas Alyamani, Feras Alshomer, Fayez Almodhen, Obaid Almeshal

**Affiliations:** 1 Plastic and Reconstructive Surgery, Surgery Department, King Abdulaziz Medical City, National Guard’s Health Affairs, Riyadh, SAU; 2 Surgery, King Abdullah International Medical Research Center, Riyadh, SAU; 3 Pediatric Urology Section, Surgery Department, King Abdulaziz Medical City, National Guard’s Health Affairs, Riyadh, SAU

**Keywords:** ectopic, scrotum, inguinal, z-plasty, flap, reconstruction

## Abstract

An ectopic scrotum (ES) represents a rare developmental anomaly of the male genitalia. The condition usually represents a part of a wide spectrum pathology associated with other anomalies. The different locations in which an ectopic scrotum is found include inguinal, suprainguinal, infrainguinal, and/or perineum. There are several surgical techniques described in the literature related to the management of ES, but none of them showed superior results. We present a rare case of ectopic hemiscrotum managed as part of a multidisciplinary team approach, showing the utility of double opposing transposition z-plasty flaps in managing such a case.

## Introduction

Various congenital anomalies that affect scrotal development might include bifid scrotum, penoscrotal transposition, accessory scrotum, or even ectopic scrotum [1]. An ectopic scrotum (ES) represents a rare developmental anomaly that usually comes with other anomalies that affect the male genitalia. ES can be in different locations, but most are usually located in the inguinal, suprainguinal, infrainguinal, or perineal area. Associated anomalies include inguinal hernia, cryptorchidism, and exstrophy of the bladder [2,3]. We present a rare case of ectopic hemiscrotum associated with hypospadias and penile torque managed by a multidisciplinary team involving plastic surgery, urology, and pediatrics.

## Case presentation

**Figure 1:**
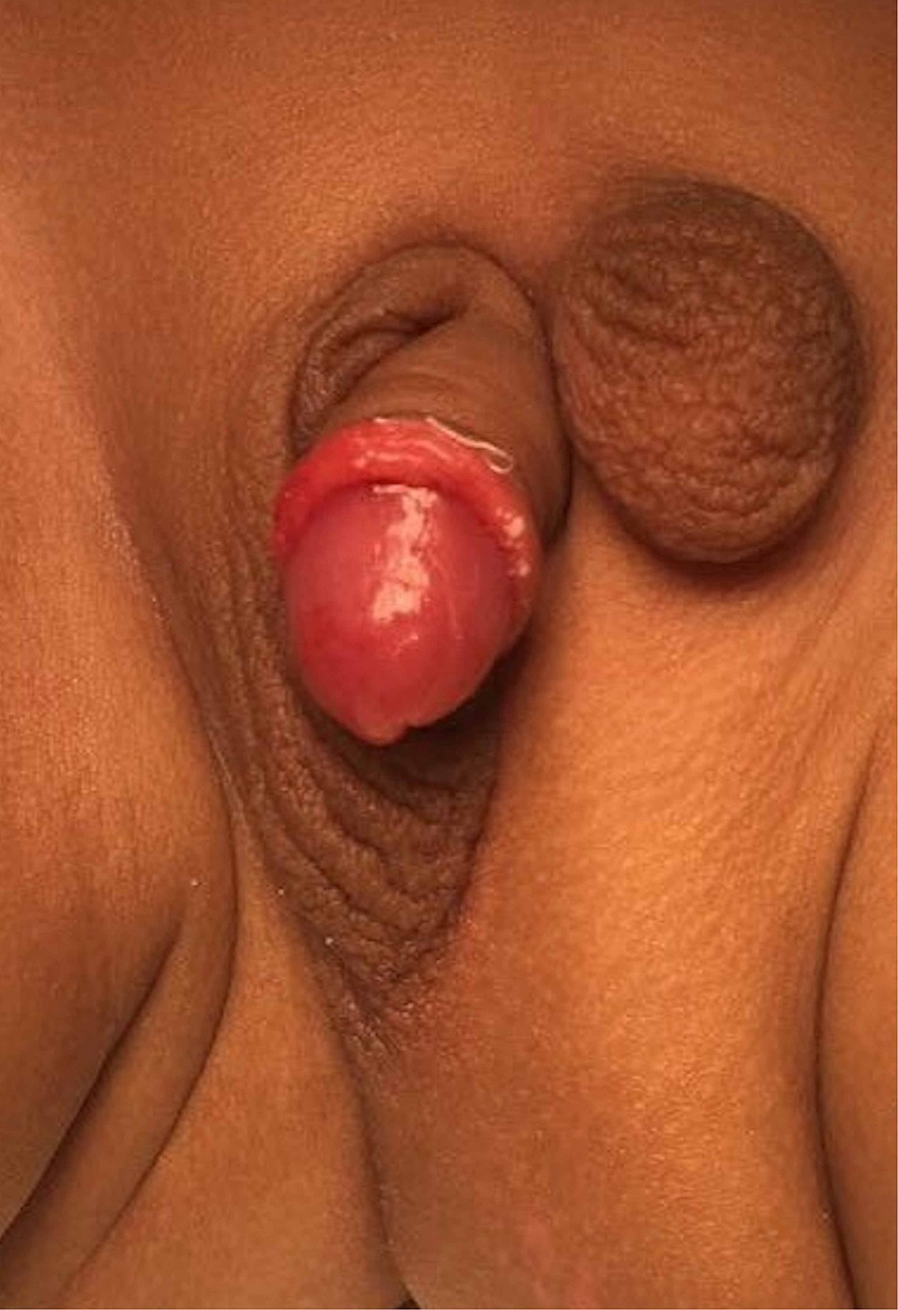
Pre-operative photo The photo shows the left inguinal ectopic hemiscrotum with associated hypospadias and penile torque.

A full-term one-month-old baby presented to the clinic with unremarkable perinatal history. Physical examination showed left inguinal hemiscrotum. The right half of the scrotum was developed normally with a normal testicle in position. The ectopic hemiscrotum was in the inguinal area with a smaller testicle but within the normal variation. He had penile torque and hypospadias (Figure 1). Renal ultrasound was normal, while cystourethrogram showed grade 1 vesicoureteric reflux with minimal opacification of the distal left ureter. Chromosomal analysis showed a normal male karyotype. 

**Figure 2:**
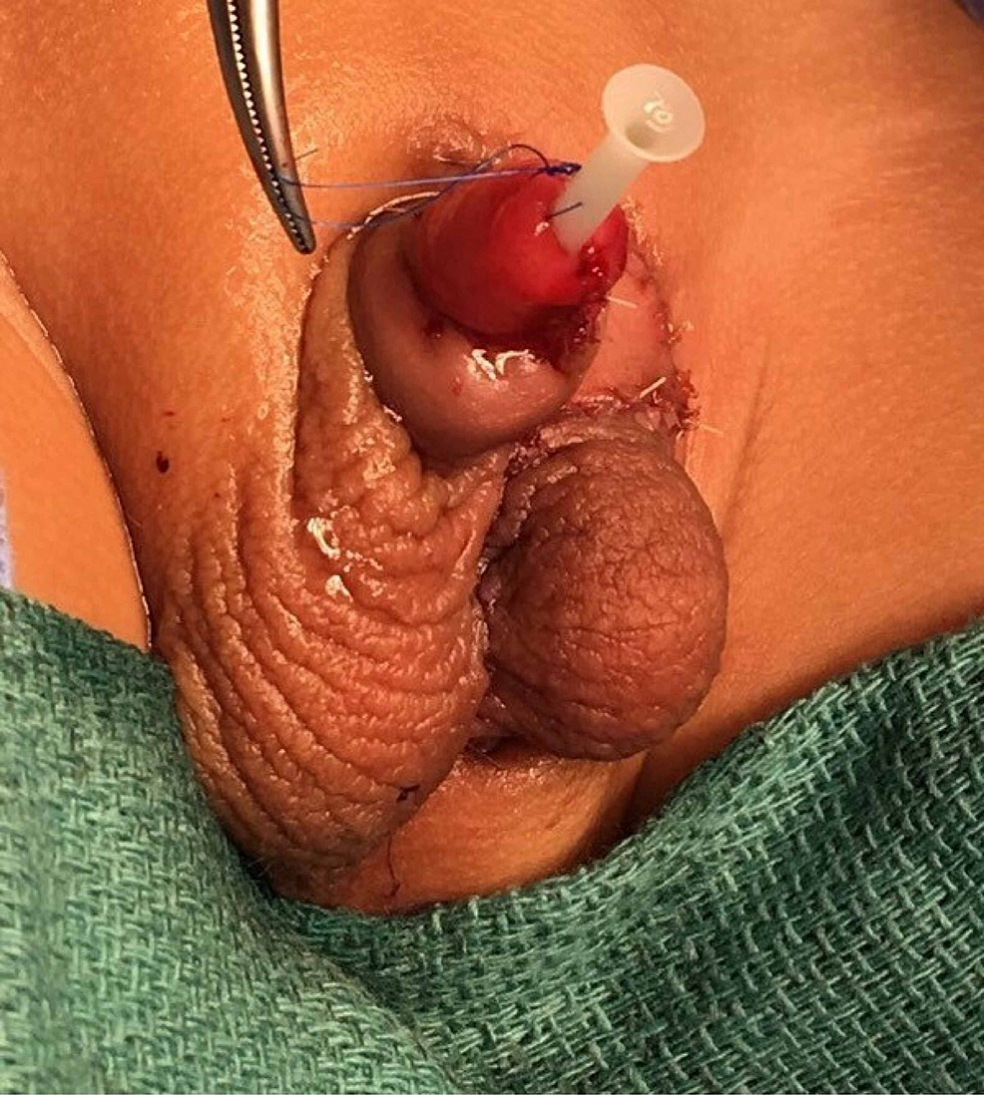
Post-operative results The photo shows the repaired ectopic hemiscrotum after block unit mobilization with the use of double opposing transposition z-plasty flap with the repair of hypospadias and penile torque, with a stent in place.

The patient was treated by a multidisciplinary team approach that involved plastic surgery, urology, and pediatrics. The patient underwent scrotoplasty, correction of the penile torque, and hypospadias at the age of thirteen months. The scrotoplasty was done utilizing the z-plasty principle with double transposition flaps. The upper flap based inferiorly containing the scrotum and the testicle was mobilized as a block after freeing the spermatic cord and then sutured to the side of the adjacent scrotum. The lower flap utilizing the skin between the two scrotii based superiorly was transported to fill the defect in the inguinal region and to minimize the penile torque deformity. The vascularity of the left hemiscrotum was maintained with no risk of testicular torsion. Subcoronal hypospadias repair was done with a stent kept place (Snodgrass hypospadias repair) (Figure 2).

**Figure 3:**
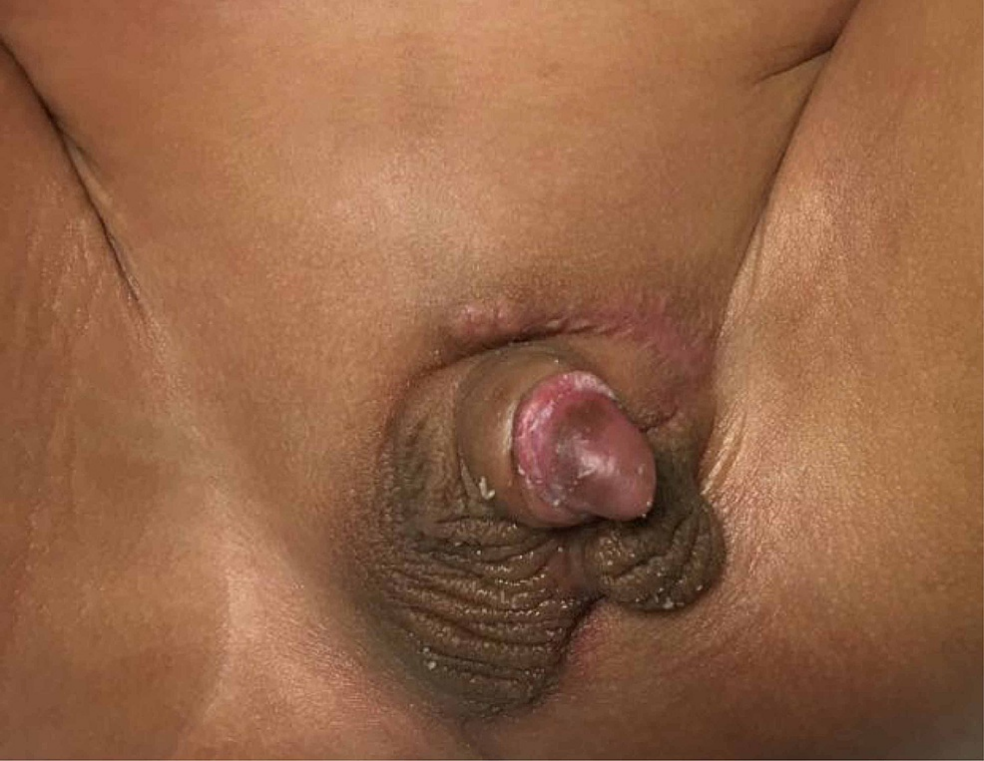
Follow-up assessment The photo shows the results after two months follow-up, with a satisfactory position of the mobilized ectopic hemiscrotum.

The patient was followed up in the clinic with a good surgical outcome, no complications, and the parents were satisfied with the result (Figure 3). The patient’s parents consented to share the information related to this manuscript.

## Discussion

The development of the scrotum is well explained in the literature. However, scrotal anomalies are quite ubiquitous. A brief explanation of the normal development of the scrotum starts with an appearance of two labiosacral swellings at four weeks of gestation lateral to the cloacal membrane. At week 12, the two labiosacral swellings grow and fuse to form the scrotum. The line along the fusion is called the raphe mediana. At the same time, the gubernaculum plays a major role in the descent of the testis by inserting into the labiosacral swelling [4]. However, the embryological explanation of the ectopic scrotum is not known. In this case, the testis was misdirected into the inguinal area with a normal variant testis. 

Reviewing the literature shows that inguinal ectopic scrotum is mostly associated with other developmental anomalies that may include and are not limited to cryptorchidism, renal agenesis, penile torsion, hypospadias, renal dysplasia, and testicular atrophy [5]. In our case, the patient, as mentioned, also had associated hypospadias and penile torque, which were corrected in the same procedure with a satisfactory outcome.

Different surgical methods have been described in the literature related to the management of ES, as summarized in (Table 1). Briefly, such techniques might include excision of the ectopic hemiscrotum with mobilization of the testis into the opposite hemiscrotum as described by Elder and Jeffs (1982) and Guha (1979) [6,7]. Sobral Filho et al. described an inverted Y incision that utilizes the perineal skin flaps in the scrotal sac reconstruction [8]. Lamm and Kaplan described a method in which the ES was brought into the normal position and sutured to the lateral aspect of the normotopic scrotum by a rotation flap [9]. The utility of z-plasty in scrotoplasty was sparsely evaluated in recent literature, in which most of its use was in a staged approach to correct any residual associated deformities [10]. In this case, we showed the great potential utility of double opposing transpositional flaps with the mobilization of the ectopic scrotum as a whole block with a great satisfactory outcome. 

**Table 1:** Summary of different surgical techniques described in the literature that are related to the management of ES

Author	Patient	Repair Technique	Outcome
Lamm 1977 [9]	Case 1: ectopic left inguinal scrotum with a deviation of the penis to right. Case 2: ectopic right inguinal scrotum as well as normotopic scrotum containing palpably normal testes.	The ectopic scrotum was used for skin coverage of the penis by rotating it down to the midline.	Wound infection with later skin contracture. Staged reconstruction was performed with the utility of z-plasty to correct the scarring at the base of the penis and to reposition the deviated penis.
Guha 1979 [7]	Congenital absence of the left half of the scrotum with a small sac of scrotal skin in the left inguinal region.	Excision of the ectopic hemiscrotum with mobilization of the testis into the opposite hemiscrotum	Not mentioned
Elder 1982 [6]	Supra-inguinal ectopic scrotum	Excision of the ectopic hemiscrotum with mobilization of the testis into the opposite hemiscrotum	Not mentioned
Gunaydin 1997 [11]	Right side: inguinal ectopic scrotum. Left side: in normal position. Each hemiscrotum was containing testis.	A pedicled flap raised in between the ectopic and normal scrotum, then it was mobilized laterally and sutured to the ectopic scrotum lateral side.	No complication
Daniel 2015 [12]	Right inguinal ectopic scrotum containing the testis. The ectopic scrotal skin had a small central skin tag. There was intervening normal skin between the two halves of his scrotum.	rotation flap	No complication
Sobral Filho 2017 [8]	Bilateral cryptorchidism with ectopic penis and scrotum found in perineal area.	Scrotal skin flaps in the perineum were utilized in the reconstruction of scrotal sac with the utility of inverted Y shaped incision	No complication
Hisamatsu 2019 [13]	Right infrainguinal hemiscrotum with testis within the sac.	Rotation flap scrotoplasty	No complication

Despite the many techniques described to deal with an ectopic scrotum, there was no definitive technique superior to others. The unique aspect of this case is that the scrotoplasty approach was different than the usual approach; here, the whole scrotum and the testis were mobilized as a block, as previously explained. Further, follow-up showed a good outcome with an acceptable functional and aesthetic result.

## Conclusions

An ectopic scrotum (ES) represents a rare developmental anomaly of the male genitalia. Such a condition is usually accompanied by other congenital defects such as hypospadias and penile torque, as seen in our case. We described a reconstructive technique that utilizes local tissue rearrangement with the utility of double opposing transposition z-plasty flaps to reconstruct the malpositioned scrotum with an excellent functional outcome with long-term follow-ups.
